# Hampstead Hospital Biennial Festival Dinner

**Published:** 1902-06-07

**Authors:** 


### HAMPSTEAD HOSPITAL BIENNIAL FESTIVAL DINNER.

Over 100 guests, among whom were the Mayor and the Member for Hampstead and three other M P.'s, sat down to dinner at the Grand Hotel, on Thursday, 29th ultimo, on the occasion of the Biennial Festival of the Hampstead Hospital.

The Hon. Rupert Guinness, C.M.G., presided, and the substantial result of the gathering was that Mr. Owthwaite, the genial honorary secretary, was able to read out a list of contributions to the funds of the institution, headed by a gift of £100 from the chairman himself, with a grand total of £1,367. This, and the increased support which wider knowledge of their needs is bound to bring, should prove of material assistance to the management in their efforts towards the erection of the long contemplated new building in a manner worthy of the borough and of the western half of north London.

Proposing the toast of the evening, "Prosperity to the Hampstead Hospital," the chairman said the most eloquent appeal a hospital could have was its own very good work. That work in their case was increasing. In 1901 they had 375 in-patients, as against 312 in the previous year, and their out-patients numbered 3,965, as compared with 2,670. It scarcely needed pointing out that more work meant increased expenditure, and though there had been an increase of income, thanks largely to the Ladies' Association, yet their income did not cover their expenditure, and the committee therefore looked to that biennial dinner for assistance in meeting their wants. The population of Hampstead was 80,000, and rapidly increasing. It was impossible to treat sickness at home so well as with the trained staff and scientific appliances available at a hospital, and there were also the many cases of accident and casualties to consider. It was satisfactory to know that their opinion that a local hospital was needed was shared by those capable of taking the widest expert view, as was shown by the support they were receiving from King Edward's Fund and the Hospital Sunday Fund. As to the new building, he was able to announce that the architect's plans had been approved. The necessary specifications were being got out, and in due course tenders for the work would be invited. About £11,000 had been promised so far towards the cost, £4,000 could be left on mortgage, and there would thus be some £17,000 to be raised during the two years that the building would be going on.

### Contrast between Charitable and Military Hospitals.

He had seen the plans and thought they looked very nice, and though he had had some personal experience of hospitals they were of a different character from this. He referred to those in South Africa, where he was for seven months; and though the army hospitals were as good as possible under the circumstances, they were very much hampered by red tape. They had to depend for their requirements not upon subscribers but on the filling up of endless blue papers, apparently designed with the express intention of preventing the doctors from giving their patients what was required. It took three boards of officers and two committees of inspection before sixpence could be spent. Then, again, army officers could not face the odium of trying to force a superior to move the War Office about something he knew to be necessary. None of that sort of thing applied in civil life, and it was not counted against a hospital that it endeavoured to keep its needs before the public. In concluding his remarks he appealed to them not to strangle such a humane effort as was being made for want of funds, and coupled with the toast the name of Mr. Malcolm, the treasurer.

### Speech by the Hon. Treasurer.

Mr. Malcolm, on behalf of all connected with the hospital, thanked the chairman most heartily for his stirring appeal, which he hoped had carried conviction not only to their heads but to their pockets also. He hoped that whatever sum they had intended to give they would immediately proceed to double it. That dinner was their only means of filling up the gap between their income and their expenditure, and he hoped that the sympathies of a wider circle also had been enlisted on behalf of the new building scheme. Their work at present was carried on under great difficulties, the premises being quite unsuitable for it. They had had a great deal of trouble in finding a site, but that had at length been overcome, and the site chosen had been greatly improved by the exchange of a portion of it they had been able to effect with the Metropolitan Asylums Board so as to give them a better frontage. The exchange had had to be arranged with the Local Government Board, which, though extremely fair, seemed also to be exceedingly slow, so that it was only a few months ago they had entered into possession. They had selected as their architect Mr. Keith Young, well known as the head of his profession in that particular branch, and, as the chairman had told them, the plans had been approved and the specifications were being prepared. But the builders would require to be paid, and they wanted to see their way tolerably clear before proceeding too far. Hitherto they had been very fortunate. One good friend in particular had promised that if they got £7,000 he would give £3,000 to make it up to £10,000; and though he was not exactly authorised to say so, yet he believed that that same friend would, if they again named a certain sum, be willing to help them as before. Inquiries throughout the country showed that the proportion of hospital beds to population varied in different towns from 1 per 1,000 to 6¼ per 1,000. At the lowest rate, their population in Hampstead being 80,000, they should provide 80 beds, and the numbers were so rapidly increasing that they ought to make it 100. However, they were only asking for 50, but they had so arranged it that they could extend the building so as to accommodate 100 beds at a further expense of only from 50 to 60 per cent. of the first half. In their present premises they had a number of patients who paid 12s. a week, and this was a class that required the utmost consideration. It was a great thing to find a man willing to pay what he could, and as far as their means would allow they would desire in the new hospital to arrange for this class as well as possible.

### Hospitals and Medical Science.

Mr. H. C. Richards, K.C., M.P., proposed the toast of the medical staff, who he said were the backbone of any hospital. From the humblest young doctor in a hospital to

DAMAGE

the greatest expert he found they were more zealous for work than eager for fees. The whole world was benefiting by the enormous progress that had been made in surgery of late years, and this progress had been made possible because they had encouraged and strengthened the hands of the hospitals, whose medical staffs had brought it about, and whose first object and first desire in life was the relief of suffering.

Mr. Edward Owen, F.R.C.S., and Dr. Heath Strange acknowledged the toast, after which Mr. Benjamin Lyon proposed the health of the council, treasurer, and hon. secretary, laying special emphasis on the services rendered by Mr. Malcolm and Mr. Owthwaite. In this he was echoed by Mr. McMillan, who spoke of Mr. Malcolm as the moving spirit of the council, whose lasting gratitude Mr. Owthwaite had earned by his invaluable assistance.

Compliments to the Chairman by his worship the Mayor of Hampstead brought the proceedings to a close.

